# CACSV: a computational web-sever that provides classification for cancer somatic genetic variants from different tissues

**DOI:** 10.1186/s12859-023-05207-1

**Published:** 2023-03-15

**Authors:** Nahla AlKurabi, Ahad AlGahtani, Turki M. Sobahy

**Affiliations:** 1 Information Systems Department, King Abdulaziz University (KAU), 7393 Jeddah, Kingdom of Saudi Arabia; 2 Research Center of King Faisal Specialist Hospital and Research Center-Jeddah (KFSHRC-J), 21499 Jeddah, Kingdom of Saudi Arabia

**Keywords:** Cancer, Somatic, Genetic variants, Classification

## Abstract

**Background:**

Understanding the role and function of genetic variants is extremely important when analyzing and interpreting a myriad of human disease processes. For cancer in general, cell-specific genetic variants are ubiquitous and distinct tissues have significantly heterogenic genetic profiles. In clinical practice, only a few genetic variants have identifiable clinical utility. Finding clinically relevant genetic variants constitute a challenging process. In addition, there had been no reference protocol to provide guidance for cancer somatic genetic variants classification and interpretation. In 2017, the first version of a reference protocol was published by the Association for Molecular Pathology, the American Society of Clinical Oncology, and the College of American Pathologists. Previously, we incorporated the reference protocol into a computational method to expedite the process of identification of clinically relevant genetic variants. In this work, we developed a computational web-server to increase the accessibility and availability of clinically relevant genetic variants.

**Results:**

Our work provides the clinical classification for ~ 3 million cancer genetic variants that are now publicly available in a shareable database on GitHub. We have developed a graphical user interface for the database to enhance the accessibility and ease-of-use.

**Conclusion:**

CACSV provides an open-source for about 3 million cancer tissue-specific genetic variants with their assigned clinical annotations.

## Background

Hundreds of thousands of genetic variants have been associated with single gene disorders, multi-factorial diseases, and cancers. Knowing the clinical annotations and the classification of common and rare genetic variants is important to carry-through precision medicine [1]. Cancer is a heterogeneous disease that manifests distinct genetic and molecular characteristics in different tissues. Cell-specific genetic variants (somatic) could elucidate the molecular functions for cancer driver genes [2]. However, the clinical classification of somatic genetic variants is further affected by the heterogeneous process of creating and using many different classification systems from well-established laboratories [3]. In an effort to manage the complexity of the analysis, the Association for Molecular Pathology (AMP), the American Society of Clinical Oncology (ASCO), and the College of American Pathologists (CAP) developed the first comprehensive algorithm to classify cancer somatic genetic variants (in 2017). The somatic genetic variants are assigned to one of four tiers; variants with strong clinical significance, potential clinical significance, uncertain significance, or benign/likely benign. The classification mostly relies on the availability of clinical and functional evidence in medical professional guidelines and literature, and on the detection of the mutations in small or large cancer screening studies [3].

We incorporated the clinical recommendations into a new computational method as described previously [4]. The National Comprehensive Cancer Network Clinical Practice Guidelines in Oncology (NCCN Guidelines^®^) were used as the source of the practical clinical evidence. Data about actionable genetic variants was extracted from the guidelines and reviewed for somatic genetics variants. In some part of the NCCN guidelines, the genetic variants description was not specific. For example, MET exon 14 skipping mutations in non-small-cell lung carcinoma (NSCLC) are therapeutics biomarkers for Crizotinib. Such “generally” described mutations were curated by acquiring information about their experimental validation available in literature, expert-reviewed databases, or oncogenicity predictions scores (like intOgen & CScape). The Precision Oncology Knowledge Base (OncoKB) was used to collect information about the drug-ability of the genetic variants. The evidence from the literature was measured by available information in COSMIC and cBioPortal databases. The level of a gene-in-tissue involvement was calculated through the data availability in the Cancer Gene Census (CGC) and the Candidate Cancer Gene Database (CCGD).

Our method was evaluated on a subset of manually reviewed variants and showed a balanced performance on a significantly imbalanced subset [4]. We collected cell-specific mutations from oncology genetic hubs, then filtered germline genetic mutations using the gnomAD database returning around ~ 3 million cancer mutations. The genetic mutations were then analyzed by the new algorithm producing AMP-ASCO-CAP categorized mutations. The classified data-sets were made available in JSON file format.

To facilitate the use of the classified database for non-bioinformatics users, we created CACSV as an online tool that shows the clinical annotations and classification for cancer mutations along with the source of evidence and any associated approved or investigational drugs.

## Implementation

### Design and execution

The graphical interface and computational engine were developed by using PHP, HTML, and JavaScript. The implementation was carried out by the cross-platform web server application *XAMPP*. The online tool was designed to accept queries by either gene name, the human transcript identification number, or the amino acid mutation description along with selecting a single type of cancer tissue or tissue of origin (11 types altogether).

### Data-sets structure

In our original work, we covered ten types of tumors including: breast, central nervous system (cns), colon, esophageal, gastric, melanoma, non-small cell lung cancer (nsclc), pancreas, rectal, and small cell lung cancer (sclc) [4]. About three million cancer mutations that are available on COSMIC, intOgen, or the bulk dataset of cBioPortal were collected and classified as previously described. Each tissue type has a table size of 21 × 2,952,170, and each table has a MySQL table. (Table 1).

**Table 1:** Database columns information

Gene	Gene name
ENS	Ensembl transcript identification number
cDNA	Coding DNA variant nomenclature
AA	Protein variant nomenclature
Tier	AMP-ASCO-CAP assigned class
Level	Level of evidence (A, B, C, or D)
Source	Source of the evidence
Bio type	Biomarker type (diagnostic, prognostic, or therapeutic)
Submitted type	Tumor site
NCCN drug	Described drug(s) in the NCCN guidelines
NCCN response	Drug response (resistant or sensitive) in the NCCN guidelines
NCCN condition	Drug related conditions in the NCCN guidelines
NCCN panel	NCCN panel recommendations
FDA for same	FDA-approved drug(s) for the submitted tissue
FDA for other	FDA-approved drug(s) for other tissues
Investigational for same	“Experimental” drugs (in humans) for the same tumor tissue
CT title	Available clinical trials
Status	Clinical trials status (recruiting, suspended, etc..)
Location	Clinical trial location
Preclinical for same	Compelling biological evidence in the same tumor site
Preclinical generic	FDA-approved drug for more than one tumor site
AC	Allele Count in COSMIC

### Classification algorithm

Cell-specific genetic variants are classified based on the availability and on the level of knowledge in medical professional guidelines, literature, mutation prevalence in the genetics databases, and gene-tissue association. Tier I mutations may be delineated in the medical guidelines, they may have fully approved drug(s), or they may be found in PanCan studies in the specified type of tissue. Tier II mutations could have an approved drug(s) for different types of cancer tissues or they could have investigational drugs or preclinical evidence. These mutations may have also been mentioned in small studies. Tier III genetic variants are outlined in a few studies in a different type of tumor tissue and have no reliable experimental functional evaluation.

## Results

**Fig. 1:**
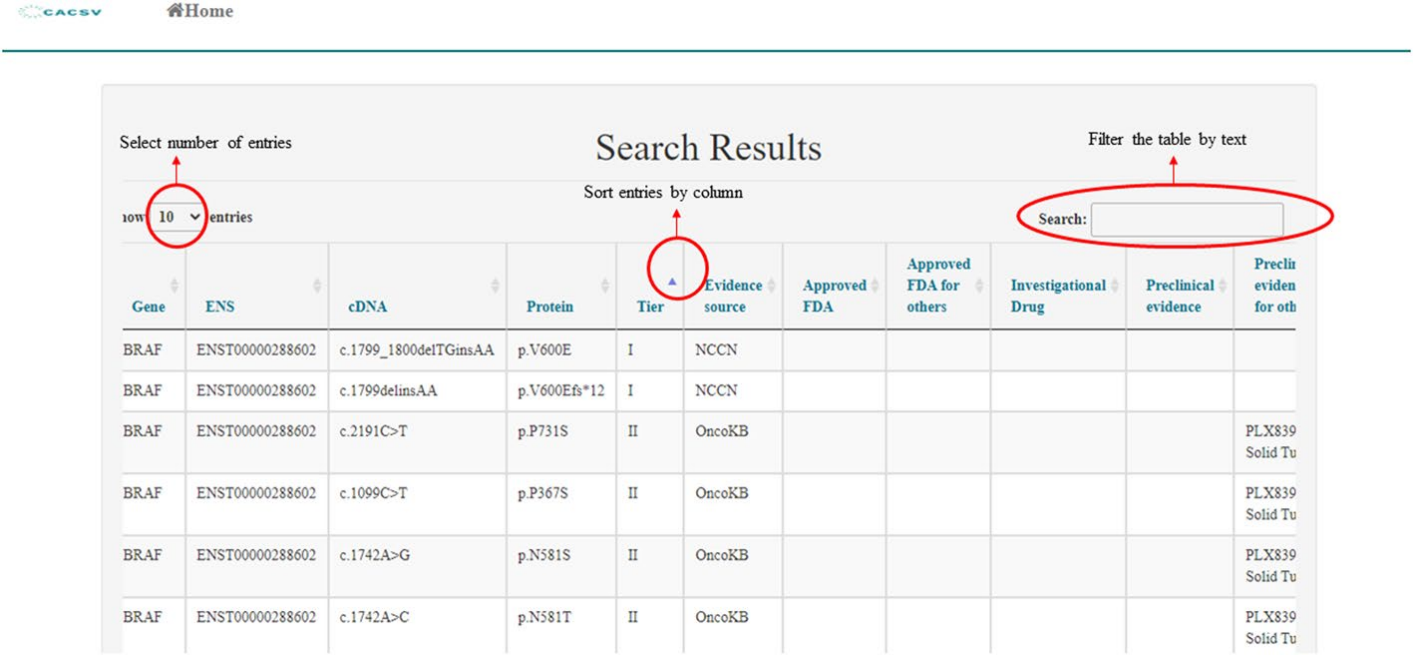
The search page is marked to show the multiple options to filter and sort the output table

The web-server can take different types of queries from users (gene name, human transcript identification number, or amino acid mutation) with one type of tissue and will return the clinical classification in a formatted table. The table presents different types of information that include the clinical classification (tier), available drugs or clinical trials, and source of evidence. The user may search the full content by string-based search through a filtering box and sort the table by any column (Fig. 1). The users can also choose the number of displayed entries per page: 10, 25, 50, or 100. Data per gene can be exported as well.

## Discussion

The number of genetics and genomics applications in oncology are immerse and the associated clinical yield is improving. Multiple research and clinical groups work on the genetic information curation and revision process. In the realm of clinical practice, the decisions of the cancer genetics variants “serviceability” to the patients are made through meetings and discussions by the tumor molecular board (TMB). Computational methods providing clinical annotations for thousands of cancer somatic genetic variants can assist in the aforementioned process. Our web-application would provide an open-source to clinical geneticists and medical oncologists during the board meetings.

Future improvements for the software are warranted. The new released clinical standards by the Clinical Genome Resource (ClinGen), Cancer Genomics Consortium (CGC), and Variant Interpretation for Cancer Consortium (VICC) may further improve the clinical annotations [5]. From technical prospective, the software could be also made easy-to-integrate with the existing sequencing analysis workflows and systems.

## Conclusion

CACSV is an easy-to-use computational method that produces clinical annotations for about 3 million cancer tissue-specific genetic variants.

## Data Availability

Data sharing is not applicable to this article as no datasets were generated or analyzed during the current study. Project name: Clinically Actionable Cancer Somatic Variants (CACSV). Project home page: http://44.203.161.191/cacsv/. Operating system(s): Platform independent. Programming language: PHP, HTML, MySQL, and JavaScript. Other requirements: update to date web-browser. License: MIT. Any restrictions to use by non-academics: license needed.

## Abbreviations

GUI: Graphical user interface
AMP: The Association for Molecular Pathology
ASCO: American Society of Clinical Oncology
CAP: College of American Pathologists
CACSV: Clinically actionable cancer somatic variants
COSMIC: Catalogue of somatic mutations in cancer
CNS: Central nervous system
ACMG: American College of Medical Genetics
